# Transfusion-Associated Graft-Versus-Host Disease in Adults

**DOI:** 10.7759/cureus.44148

**Published:** 2023-08-26

**Authors:** Erwa Eltayib Elmakki, Mohammed Ali Madkhali, Omar Oraibi, Sameer Alqassimi, Eman Saleh

**Affiliations:** 1 Department of Internal Medicine, Faculty of Medicine, Jazan University, Jazan, SAU

**Keywords:** prevention, diagnosis, pathophysiology, risk factors, ta-gvhd

## Abstract

Transfusion-associated graft-versus-host disease (TA-GVHD) is a rare but fatal complication of blood transfusion that usually develops two to 30 days following a blood transfusion giving rise to graft versus host disease (GVHD) clinical features that are consisting of fever, skin rash, jaundice, diarrhea, and pancytopenia. The disease is fulminant in most patients with a mortality rate of >90% of cases. The main aim of this review is to enhance awareness among medical practitioners about this fatal disease. Data were extracted manually from the main medical databases (Medline, Scopus, and Google Scholar) after the revision of selected articles and assessed for their contribution to the knowledge of TA-GVHD.

TA-GVHD occurs when the viable donor T-cells in the blood or blood products attack the recipient's tissues which his/her immune system is incapable to destroy due to several reasons. The recipient's tissues that are usually involved in TA-GVHD include the liver, intestine, skin, lungs, and bone marrow. Any blood component either whole blood, packed red blood cells (RBCs), platelets, or fresh non-frozen plasma that contains viable T lymphocytes can cause TA-GVHD. Host immunodeficiency, transfusion of fresh blood, and partial human leukocyte antigen (HLA) matching between the donors and the recipients represent the major risk factors of TA-GVHD. Partial HLA matching includes immunocompetent recipients who receive blood from a first-degree relative also, seen in genetically homogenous populations because of high rates of consanguineous marriage. The diagnosis of TA-GVHD is mainly suspected based on clinical manifestations. However, a histopathological study of either skin or rectal biopsy is diagnostic. The treatment of TA-GVHD is generally not effective, unless the patient received emergency stem cell transplantation, while prevention via irradiation of blood or blood products represents the standard of care for this disease.

In conclusion, medical practitioners should have a high index of suspicion for this disease. Moreover, future clinical trials targeting and comparing the outcomes of the different therapeutic options for TA-GVHD are required.

## Introduction and background

Blood transfusion (BT) is one of the widely used medical procedures in clinical practice which help to treat many conditions, especially in hospitalized patients. Hence, it is crucial that clinicians should be aware of BT complications and their clinical manifestations [1]. Transfusion complications are anything that occurs during or after a BT. BT complications can be categorized as acute which is defined as the ones that occur during or within 24 hours of BT while those that occur more than 24 hours from the end of BT are called delayed complications. One of the fatal delayed complications of BT is transfusion-associated graft-versus-host disease (TA-GVHD) [2]. Graft versus host disease (GVHD) is a condition that can occur after hematopoietic stem cell transplantation (HSCT). It happens when the transplanted T-cells recognize the recipient's body as foreign and attack it [3]. Solid organ transplantation, particularly such as that of bone marrow or stem cells, and transfusion of non-irradiated blood or blood products are the major risk factors of GVHD [4]. In TA-GVHD, the donor T-cells (T lymphocytes) in a transfused blood or blood product attack the recipient's tissues including the skin, intestine, liver, and bone marrow [1,5-7]. TA-GVHD usually occurs 5-10 days after the process of BT. However, symptoms of TA-GVHD can develop as early as three days and as late as 30 days [1,5]. TA-GVHD is fatal in more than 90% of cases due to aplasia of the recipient's bone marrow, leading to infection and hemorrhage [5-7]. TA-GVHD can develop after transfusion of whole blood, red blood cells (RBCs), platelets (including human leukocyte antigen [HLA]-matched), granulocytes, and fresh unfrozen plasma [8]. The literature search revealed only one systematic review of TA-GVHD. It was conducted in 2015 by Kopolovic et al. Their study included 348 cases of TA-GVHD. They found that 38.2% of cases were caused by RBC transfusion, 26.4% were due to whole BT, 5.7% were caused by platelet transfusion, 0.6% were caused by buffy-coat product transfusion, 0.3% were caused by plasma transfusion, and 28.7% were caused by non-identified blood components [8]. Clinical manifestations of TA-GVHD are similar to that of GVHD caused by bone marrow transplantation. They include erythematous, maculopapular skin rash, fever, elevated liver enzymes, hepatomegaly, and jaundice, in addition to nausea, vomiting, and diarrhea. However, in TA-GVHD, the onset of these symptoms occurs earlier than in GVHD associated with stem cell or bone marrow transplantation. Furthermore, bone marrow aplasia is more severe in TA-GVHD. Because TA-GVHD results in profound bone marrow failure, the prognosis of it is very poor [9]. TA-GVHD is a clinical diagnosis and should be suspected if the recipient develops a fever, skin rash, and pancytopenia following a BT. The diagnosis of TA-GVHD is similar to GVHD caused by bone marrow transplantation or HSCT. Biopsy from the skin (most commonly), liver, or bowel is diagnostic for TA-GVHD [9].

There is no effective treatment for TA-GVHD. However, this disease can be prevented effectively by giving irradiated blood products, particularly for at-risk individuals [10].

The diagnosis of TA-GVHD can easily be missed in clinical practice due to the lack of awareness of this condition in addition to its similarity with various other medical conditions. Thus, the main aim of this review is to raise awareness about this fatal complication of BT by discussing its risk factors, pathogenesis, clinical presentation, diagnosis, and prevention.

## Review

Incidence

According to recent reports, TA-GVHD is a rare complication of BT, and its exact incidence is not known as this depends on the population receiving the transfusion [11]. However, the incidence of GVHD after an allogeneic bone marrow transplant is common and approximately reaching 50% of cases. On the other hand, the incidence of TA-GVHD according to some studies ranges between 0.2% and 5%, and generally, it depends upon the studied population [10]. In Saudi Arabia, a literature search revealed no published data on the incidence of TA-GVHD. However, a recent study by Chentoufi and colleagues revealed a high prevalence of homozygosity of HLA class I and class II alleles and haplotypes in the Saudi population, which reflects the degree of genetic diversity (or similarity) in the studied population [12]. The incidence of TA-GVHD was common in Japan, which is attributed to the high levels of consanguinity in that country. This possibly leads to widespread HLA matching among the population, so this is why there is the universal use of irradiated BT in that country [10]. TA-GVHD is most seen in immunosuppressed individuals, such as those with hematologic malignancies, and patients who receive bone marrow or solid organ transplants. Interestingly, the incidence of TA-GVHD is low among individuals with HIV infection. TA-GVHD is also more likely to occur in patients who receive blood or blood products from first-degree relatives such as siblings or other related donors [13-15].

Risk factors

The three main risk factors for TA-GVHD are the volume and pattern of the blood transfused as this indicates the number of viable T cells in the transfused blood; HLA haplotype sharing between donor and recipient; and the depressed immune function. Immunodeficiency either primary or secondary can inhibit the capability of recipients' immune system to damage donor T-cells. Examples of immunodeficiency conditions that can precipitate the development of TA-GVHD include old age, the use of immunosuppressive medications, hematological malignancies, and bone marrow transplantation or HSCT [8,11,13,16-22]. Interestingly, this is not applied in patients with HIV infection, and hence HIV infection is not considered a risk factor for TA-GVHD. This might be attributed to the fact that the donor T-cells become infected with HIV, so will not be able to attack the host tissues [9].

Other risk factors are transfusion of fresh blood, and failure to adopt preventive measures such as irradiation of blood products, or use of a blood filter, which removes donor lymphocytes, may increase the risk of developing TA-GVHD. Interestingly, one or more of these risk factors has been detected in most reported cases [8,11,20-22]. However, Kopolovic et al. in their study, which included 348 patients with TA-GVHD, revealed that still 50% of the studied patients had no apparent risk factors [8]. One of the important risk factors for TA-GVHD is a partial HLA matching between the donor and recipient. This typically occurs in situations in which an immunocompetent person receives blood from a relative also can be seen in genetically homogenous communities or populations as a result of high rates of consanguineous marriage (Table 1). HLA matching is important because it helps to reduce the risk of transfusion reactions, graft rejection, and GVHD [8,23,24].

**Table 1:** Risk factors of TA-GVHD. HLA, human leukocyte antigen; TA-GVHD, transfusion-associated graft-versus-host disease.

At-risk status
Immunodeficiency
Elderly persons
Multiple and prolonged transfusions
Partial HLA matching
Immunocompetent who receives blood from a relative
Consanguineous marriage
Transfusion of fresh blood products
Patients with stem cell or bone marrow transplants

Pathophysiology

GVHD mainly occurs after HSCT as the donor-viable T-cells in the blood or blood products attack antigen-presenting cells with various HLA haplotypes in the recipient tissues, particularly in the skin, liver, and gastrointestinal tract. But can we have a GVHD in non-transplant patients? In normal circumstances, BTs would not be expected to result in GVHD, as the host's immune system would remove the T-cells in the donor blood. However, GVHD has been reported after BT (TA-GVHD) in patients who have not had an organ transplant. This mainly occurs in two ways. First, the patient is immunocompromised, either because of an underlying T-cell disorder, such as the users of chemotherapy or radiotherapy for hematologic or solid organ cancer [8,10,24]. So, the donor T-cells directly attack the recipients' organs. The second way is unique and develops in immunocompetent individuals. This mainly occurs when an HLA heterozygous recipient receives a BT from a homozygous donor for that HLA antigen [23]. HLA molecules are kinds of proteins located at the surface of all the cells in the human body. They play an important role in the recognition of strange antigens by the immune system and are highly variable. As far as BT reactions are concerned, HLA matching is crucial because it helps to minimize the risk of the development of TA-GVHD [8]. Thus, when a person receives a BT from a partially HLA-matched donor, the recipient's T-cells do not recognize the blood donor’s T-cells as strange subjects; however, the donor T-cells recognize the “mismatched” HLA antigen in the host tissues. Donor cells can attack recipient tissues including bone marrow, skin, liver, and intestine. This kind of genetic interaction is likely to occur when there is limited genetic variability among the population, as in Japan, or when the blood donor is a relative, particularly if there is a high level of consanguinity, thus causing shared HLA antigens [8,25,26].

The mechanism of TA-GVHD involves the activation and proliferation of donor T-cells, which recognize and attack the recipient's tissues. The release of pro-inflammatory cytokines such as natural killer cells and chemokines by activated T-cells causes tissue damage and systemic inflammation. TA-GVHD can affect multiple organs, including the skin, liver, lungs, gastrointestinal tract, and bone marrow [9,23,24]. Unlike transplantation-associated GVHD, TA-GVHD is almost always fatal. This is because apart from attacking the skin, liver, and intestines, T-cells in the donor blood also attack the host’s bone marrow, causing bone marrow aplasia. Remember, that in HSCT, the marrow has been ablated, and replaced by the donor marrow, which will therefore not be attacked by the graft lymphocytes [9,26]. Overall, the pathophysiology of TA-GVHD involves an immune response against the recipient's healthy tissues triggered by donor T-cells, leading to severe and potentially fatal multi-organ dysfunction consisting of hepatitis, diarrhea, maculopapular skin rash, and pancytopenia due to bone marrow failure [8,10,11].

Clinical manifestations

The symptoms of TA-GVHD usually develop one to two weeks following cellular BT [11,27]. Fever is the most common symptom of TA-GVHD. It is usually associated with a generalized pruritic maculopapular skin rash commonly involving the face, trunk, and extremities.

In addition, TA-GVHD may lead to multiple gastrointestinal symptoms in the form of bloody diarrhea, abdominal pain, nausea, and vomiting [11,28]. Moreover, TA-GVHD commonly involves the bone marrow and results in thrombocytopenia or pancytopenia. Also, liver dysfunction characterized by jaundice, hepatomegaly, and elevation in liver enzymes (alanine transaminase [ALT], aspartate aminotransferase [AST], ALP [alkaline phosphatase]) can be seen in patients with TA-GVHD. On the other hand, respiratory and central nervous system involvements leading to shortness of breath, hypoxemia, aseptic meningitis, encephalitis, and seizures may occur in some patients with TA-GVHD [17,29,30]. Hence, clinically, the diagnosis of TA-GVHD should be suspected once a patient develops a fever, cytopenia or pancytopenia, maculopapular skin rash, hepatitis, and diarrhea with or without the other mentioned symptoms (Table 2), particularly if these symptoms develop a few days or weeks following a BT [27]. 

**Table 2:** Clinical features of TA-GVHD TA-GVHD, transfusion-associated graft-versus-host disease.

Clinical presentation
Fever
Skin rash
Diarrhea
Jaundice
Respiratory distress
Seizures
Hepatomegaly
Elevation of liver enzymes
Elevation of total bilirubin
Cytopenia or pancytopenia

Diagnosis

The diagnosis of TA-GVHD is usually a challenging issue as the symptoms of this condition are non-specific and can mimic many other similar diseases such as viral infections, drug reactions, autoimmune conditions, liver failure, aplastic anemia, immunodeficiency syndrome, and hematologic malignancies (Table 3) [31]. The classic clinical features of TA-GVHD include fever, skin rash, diarrhea, liver dysfunction, and bone marrow suppression [32]. However, not all patients with TA-GVHD exhibit these symptoms, and some may present with atypical or delayed manifestations. Histopathological examination of either the skin, rectum, liver, or bone marrow biopsy specimens can reveal characteristic changes suggestive of GVHD, such as lymphocytic infiltration, necrosis, and epithelial damage. Biopsy from any of the above-listed organs is the golden test for establishing the diagnosis of TA-GVHD. However, a skin biopsy is the least invasive and easier to perform in comparison with a liver or bone marrow biopsy. On the other hand, a biopsy may not always be feasible or conclusive [30,33-35]. Another test for diagnosing TA-GVHD is by demonstration of white blood cell chimerism using short tandem repeat-polymerase chain reaction (STR-PCR). This is confirmed by finding different HLA phenotypes in the recipient's lymphocytes [13,36,37]. Alternative cytogenetic tests that can be used to confirm TA-GVHD is fluorescent in situ hybridization (FISH) [15,36]. Cytometric analysis of peripheral blood can detect the presence of donor T cells in the recipient's circulation, which is potential for GVHD. The sensitivity and specificity of this test depend on the timing and frequency of sampling and the sensitivity of the markers used to differentiate donor and recipient cells [15,36]. Moreover, the FISH test is rapid and more sensitive when the donor and recipient are of a different gender [15].

**Table 3:** Differential diagnosis of TA-GVHD. TA-GVHD, transfusion-associated graft-versus-host disease.

Similar conditions
Viral infections
Drug reactions
Autoimmune disorders
Liver failure
Aplastic anemia
Immunodeficiency syndrome
Hematologic malignancies

Management

The best way to manage TA-GVHD is to prevent it from occurring [11]. The cornerstone of treating TA-GVHD is immunosuppressive therapy, which aims to dampen the donor's immune response to the recipient's tissues. High-dose corticosteroids are often used as a first-line treatment, but other immunosuppressive agents, such as cyclosporine, tacrolimus, and anti-thymocyte globulin, may also be necessary depending on the severity and progression of the disease [8]. A systematic review that involved 349 participants revealed that half of the survivors received immunosuppressive agents. However, the total number of survivors in that study was small (29 patients), which made it statistically insignificant [8]. Furthermore, immunosuppressive agents in several case reports were not shown to be effective [38,39]. However, according to the new guidelines, there is hope for using monoclonal antibodies for immunosuppression [40].

Emergency allogeneic HSCT is the most effective treatment option for severe cases of TA-GVHD. HSCT from a matched sibling donor or unrelated donor may be an option. This procedure replaces the recipient's damaged bone marrow with healthy donor cells that do not carry the HLA antigens responsible for the graft-versus-host reaction [33,41,42].

The main drawback of HSCT in TA-GVHD is the long time needed to determine an appropriate donor while the disease progresses rapidly, making it a non-viable therapeutic option [8,33].

Ruxolitinib, which is a Janus-associated kinases inhibitor (JAK1 and JAK2), was recommended by the American FDA in 2019 for the treatment of steroid-refractory acute GVHD in patients aged 12 years or above and has become a preferred option for this situation [43].

Prevention

The golden rule in medicine that says "prevention is better than cure" is totally applicable to TA-GVHD; hence, TA-GVHD is a preventable condition, whereas there is no specific effective treatment for it [33].

One of the most effective ways to prevent TA-GVHD is to use irradiated blood products. Irradiation of the cellular blood components using gamma or X-rays can kill the white blood cells including viable T-cells in the donated blood and prevent them from attacking the recipient's tissues. This is a standard procedure for certain types of BTs, such as those allocated to immunocompromised patients [10,17,37,41,44,45]. Interestingly, there is no difference between gamma and X-ray irradiation in terms of efficacy according to an in vitro study [46]. The blood products that need to be treated or irradiated include whole blood, packed RBCs, platelets, granulocytes, and non-frozen plasma while fresh frozen plasma does not need to be irradiated [8,33].

Those who are considered for chimeric antigen receptor T-cell (CAR-T) therapy should be given irradiated blood components, seven days before the harvest and continue till three months after the CAR-T cell infusion. This is according to the recent updates of the British Society of Hematology guidelines [27,42]. Another effective preventive measure includes pathogen inactivation and freeze-thaw processes prior to BT which efficiently helps to inactivate T-cells [42]. Pathogen inactivation is a technique in which the infectious agents in the blood are removed prior to BT. This technique was also found to be effective in inactivation of T-cells. In some preclinical studies, pathogen inactivation was found to be more effective than gamma irradiation in the prevention of TA-GVHD [47,48]. However, because TA-GVHD is a rare condition, still there is no adequate data among human beings comparing the efficacy of gamma irradiation versus pathogen inactivation procedures. Future clinical trials are needed in this virgin field [49]. Other preventive measure includes the use of leukocyte filters or leucodepletion (leukoreduction) for at-risk individuals. Leukocyte filters can trap white blood cells in donated blood and prevent them from causing TA-GVHD. These filters are commonly used for transfusions to high-risk patients, such as those undergoing organ transplants or receiving chemotherapy [21,42,50]. On the other hand, some reported cases by Akahoshi et al. and Hayashi et al., respectively, described the occurrence of TA-GVHD despite the use of leucodepletion techniques [51,52]. 

Prognosis

Generally, the prognosis of TA-GVHD is very poor without effective urgent treatment, and according to several studies, the mortality of TA-GVHD is >90% [9-11]. Moreover, Kopolovic et al. in their systematic review which included 348 cases of TA-GVHD reported a survival rate of only 8%. The survival rate in their study was higher in young age groups and in those who received stored blood products [8]. Furthermore, milder cases of TA-GVHD were reported and it is assumed that many milder cases can be misdiagnosed or overcome in clinical practice [23,33].

## Conclusions

TA-GVHD is an uncommon but serious complication of BT that occurs when the donor's immune cells attack multiple recipient tissues. Development of a triad of features consisting of fever, pancytopenia, and maculopapular skin rash a few days or weeks following a BT should direct the attention toward TA-GVHD and the clinicians should have a high index of suspicion for it, particularly when dealing with a high-risk group of patients. An early diagnosis of TA-GVHD is crucial for the timely initiation of appropriate treatment. Currently, urgent HSCT is the only definitive treatment. Moreover, the adoption of standard preventive measures among high-risk groups is of paramount importance.

The literature search revealed that there is a knowledge gap regarding the outcomes of immunosuppressive therapy in TA-GVHD. Hence, data on TA-GVHD in terms of clinical trials is still lacking. Thus, future clinical trials are required to compare the outcomes of the different therapeutic options for TA-GVHD.
